# New family of biosensors for monitoring BTX in aquatic and edaphic environments

**DOI:** 10.1111/1751-7915.12394

**Published:** 2016-08-03

**Authors:** Verónica Hernández‐Sánchez, Lázaro Molina, Juan Luis Ramos, Ana Segura

**Affiliations:** ^1^ Estación Experimental del Zaidín‐CSICC/ Profesor Albareda s/n18008GranadaSpain

## Abstract

Benzene, toluene, ethylbenzene and xylenes (BTEX) contamination is a serious threat to public health and the environment, and therefore, there is an urgent need to detect its presence in nature. The use of whole‐cell reporters is an efficient, easy‐to‐use and low‐cost approach to detect and follow contaminants outside specialized laboratories; this is especially important in oil spills that are frequent in marine environments. The aim of this study is the construction of a bioreporter system and its comparison and validation for the specific detection of monocyclic aromatic hydrocarbons in different host bacteria and environmental samples. Our bioreporter system is based on the two component regulatory system TodS–TodT of *P. putida* DOT‐T1E, and the *P*_*todX*_ promoter fused to the GFP protein as the reporter protein. For the construction of different biosensors, this bioreporter was transferred into three different bacterial strains isolated from three different environments, and their performance was measured. Validation of the biosensors on water samples spiked with petrol, diesel and crude oil on contaminated waters from oil spills and on contaminated soils demonstrated that they can be used in mapping and monitoring some BTEX compounds (specifically benzene, toluene and two xylene isomers). Validation of biosensors is an important issue for the integration of these devices into pollution‐control programmes.

## Introduction

Benzene, toluene, ethylbenzene and xylenes (BTEX) contamination of soils and waters is a serious threat to public health and the environment, and it is essential to detect its presence in nature and to control its biodegradation in polluted sites with reliable tools (Heitzer and Sayler, 1993; Alberici *et al*., 2002; Behzadian *et al*., 2011; Wiwanitkit, 2014). The use of whole‐cell biosensors is an efficient, easy‐to‐use and low‐cost approach to monitor contaminants in the environment (Belkin, 2003) that can be used outside specialized laboratories; this is especially important in oil spills that are frequent in marine environments in remote locations (Doerffer, 2013). The construction of bioreporters is based on a natural regulatory circuit composed of a transcription regulator and a promoter or operator which is combined with a promoter‐less gene that encodes an easily measurable protein (the reporter protein). The activation of the promoter by the transcription regulator in the presence of a specific chemical compound leads to the expression of the reporter gene. The resulting output signal can be detected, calibrated and interpreted (Van der Meer and Belkin, 2010). Environmental applications for bioreporters and their advantages have been discussed by Harms *et al*. (2006).

The aim of this study is the construction, comparison and validation of biosensors for the specific detection of monocyclic aromatic hydrocarbons in different environments. Our bioreporter system is based on the two component regulatory system TodS–TodT of *P. putida* DOT‐T1E, and the *P*_*todX*_ promoter fused to the GFP protein as a reporter protein. It was previously determined that toluene was the best inducer of the *P*_*todX*_ promoter and that this induction was controlled by the sensor kinase, TodS, that phosphorylates its cognate response regulator, TodT (Lacal *et al*., 2006; Busch *et al*., 2007, 2009; Silva‐Jiménez *et al*., 2015; Koh *et al*., 2016). For the construction of different biosensors that would be functional in different environments such as soils and fresh and marine waters, this bioreporter was transferred into three different bacterial strains that were isolated from three different environments.

## Results and discussion

### Bioreporter construction and general characteristics

**Figure 1:**
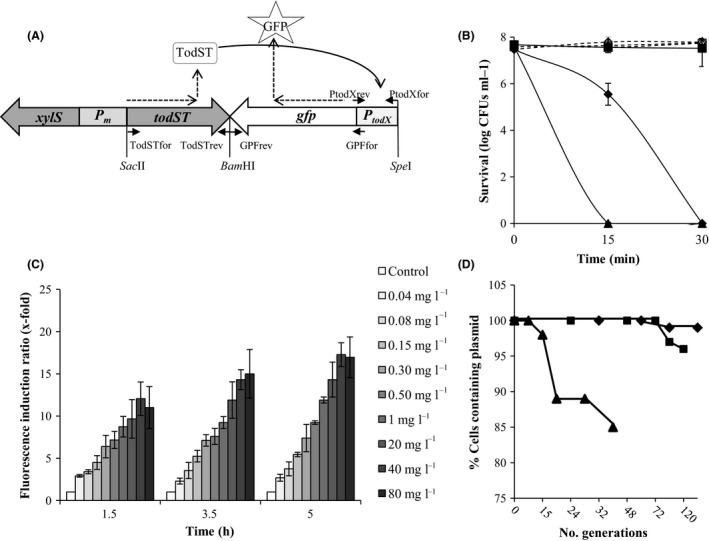
Structure and characterization of the biosensor. (A) Schematic representation of the BTX bioreporter in plasmid pKST‐1. Although the *todST* gene expression is under the control of the *Pm* promoter (inducible by benzoate or methyl‐benzoate) (Kessler *et al*., 1994), in our biosensors, basal transcription from the *Pm* promoter was high enough as to allow the induction of the system, and therefore, no benzoate or methyl‐benzoate were used in the experiments. In the presence of the effector (i.e. toluene), TodST proteins act over *P*_*todX*_ promoter inducing the expression of the GFP protein. Small arrows indicate the location of the primers used in the amplification (above/below the arrow, the name of the primer was indicated). Location of the restriction enzymes introduced in the construction, *Sac*II, *Bam* HI and *Spe*I are also depicted. (B) Solvent tolerance of the three host strains: Cultures were grown overnight and diluted to OD ≈ 0.1 next day on LB; cultures were grown until mid‐exponential phase (OD ≈ 0.8) and then divided into three flasks; 0.1% and 02% (v/v) toluene were added to two flasks and the third one was kept as control. Serial dilutions of samples taken 0, 15 and 30 min after toluene addition were drop plating to obtain the number of viable cells. Results of experiments with 0.1% toluene are not represented in the graphic. Triangles: *Alcanivorax borkumensis* SK2; diamonds: *Pseudomonas putida* KT2440; squares: *P. putida* DOT‐T1E. Discontinuous lines: samples without toluene; continuous lines: samples with 0.2% (v/v) toluene. (C) Optimum exposure time of the biosensor *A. borkumensis* SK2 (pKST‐1). Cultures were grown overnight on marine media ONR7a (Dyksterhouse *et al*., 1995) with sodium pyruvate 1% (v/v) plus streptomycin (50 μg ml^−1^) in an orbital shaker at 30°C and 200 rpm; next day the cultures were washed three times and cultivated in fresh media until they reached an OD_660 nm_ of 0.1. At this moment, different concentrations of toluene were added to each culture and samples were taken at different times. Fluorescent values are given as fluorescence induction ratio (fluorescence emitted in conditions of induction/ fluorescence emitted by the control culture without inducer). Fluorescence was measured in a LPS‐220B fluorometer (Photon Technology International). λ_ex_ was 485 nm and λ_em_ 510 nm. Error bars mean the standard deviation of three experimental repeats. (D) Plasmid stability in the three host strains: triangles: *A. borkumensis* SK2; diamonds: *P. putida* KT2440; squares: *P. putida* DOT‐T1E. Overnight cultures of the three strains grown in their corresponding media (marine media plus sodium pyruvate for *A. borkumensis* SK2 and M9 minimal media with glucose for the two *Pseudomonas* strains) plus streptomycin were diluted to OD_660 nm_ of 0.1, washed once and transferred to fresh medium without antibiotic and grown at 30°C. Serial dilutions of the culture were plated every day. The percentage of cells containing pKST‐1 plasmid was calculated as follows: (CFUs in plates with antibiotic/CFUs in plates without antibiotic) ×100. Generation times for each strain were 197 min for *A. borkumensis* SK2, 45 min for *P. putida* KT2440 and 60 min for *P. putida* DOT‐T1E.

For the construction of the bioreporter, the *P*_*todX*_ promoter and the *todST* genes were amplified using *P. putida* DOT‐T1E chromosomal DNA as template (Table S1). The GFP gene was amplified from plasmid pGREEN‐TIR (Miller and Lindow, 1997). DNA fragments *P*_*todX*_ and GFP were used as a template in an overlapping PCR to obtain a PCR fragment containing *P*_*todX*_::GFP fusion that was cloned on plasmid pSEVA438 (Silva‐Rocha *et al*., 2013) using appropriate restriction enzymes, resulting in plasmid pKST1 (Fig. 1A).

To compare performance of this construction in different strains and to be able to use the biosensor in different environments, pKST1 was electroporated into two *Pseudomonas* strains; the solvent tolerant bacteria *Pseudomonas putida* DOT‐T1E isolated from a wastewater treatment plant (Ramos *et al*., 1995) and the soil and root colonizer *P. putida* KT2240 (Nakazawa, 2002); and conjugated as in Sabirova *et al*. (2006) in the marine strain *Alcanivorax borkumensis* SK2 (Yakimov *et al*., 1998). Selection of the transformants was done using 150 μg ml^−1^ of streptomycin for the two *P. putida* strains and 50 μg ml^−1^ of the same antibiotic for *A. borkumensis* SK2.

BTEX are highly toxic for microorganisms. To compare the solvent tolerance of the three strains, we used toluene as a model compound in the determination of the survival rate of a culture after the sudden addition of 0.1% and 0.2% (v/v) of this compound. *P. putida* DOT‐T1E was described as solvent‐tolerant strain (Rojas *et al*., 2001) and, as expected, 100% of the cells survived the shock with 0.1% (not shown) and 0.2% of toluene (Fig. 1B). *P. putida* KT2440 was originally isolated from soil and has been classified as a medium‐low solvent tolerance strain (Segura *et al*., 2003; Rodríguez‐Herva *et al*., 2007). One hundred per cent of the cells in the culture survived the sudden addition of 0.1% of toluene, but only 10^6^ cells ml^−1^ were still viable 15 min after the shock with 0.2% of toluene, and after 30 min, the number of colony‐forming units (CFUs) was below the limit of detection (Fig. 1B). Information regarding *A. borkumensis* SK2 tolerance towards some alcohols and phenols was available (Naether *et al*., 2013), but there was no information about the toxicity of BTEX compounds. Cultures of *A. borkumensis* SK2 survived the 0.1% (v/v) toluene shock after 30 min; however, 15 min after 0.2% (v/v) toluene shock, the number of CFUs was below the limit of detection (< 10^2^ cells ml^−1^) (Fig. 1B). Therefore, we concluded that *P. putida* KT2440 and *A. borkumensis* SK2 were able to tolerate only moderate concentrations of toluene; this is an important issue as detection of toxic compounds by biosensors needs to take into account the concentration range in which cells are viable.

To study biosensor performance, we first determined the optimum exposure time to establish the magnitude of the response of the bioreporter to different concentrations of the target compounds (Hynninen *et al*., 2010). Although there were no significant differences in induction rates at low toluene concentrations between exposition time of 3.5 and 5 h, at high toluene concentrations, the relative fluorescence induction ratio was highest after 5 h in *A. borkumensis* SK2 (pKST‐1) (Fig. 1C). Similar induction times were obtained for *P. putida* KT2440 (pKST‐1) biosensor (data not shown). Although we did not determine the optimum exposure time for the reporter in *P. putida* DOT‐T1E, Lacal *et al*. (2006) had previously determined that the optimum induction time for the reporter system using a *P*_*todX*_ *::lacZ* fusion in *P. putida* DOT‐T1E was also 5 h; therefore, we considered this exposure time as the optimum for the three biosensors. The induction time of the three biosensors was low enough to represent an advantage compared to the determination of compounds by chemical methods.

Plasmid pKST‐1 was very stable in the absence of antibiotics in the two *P. putida* strains; 100% of the cells bore the pKST‐1 plasmid after 72 generations (Fig. 1D). In *A. borkumensis* SK2, only 85% of the cells maintained the plasmid after 40 generations (Fig. 1D). As the number of generations after 5 h (biosensor induction time) was 1.5, 6 and 5 for *A. borkumensis* SK2, *P. putida* KT2440 and *P. putida* DOT‐T1E, respectively, we can conclude that during the course of the experiment, there were no significant plasmid losses that could represent a decrease in the fluorescence measurements.

### Determination of detection and saturation limits for toluene and other BTEX compounds

**Figure 2:**
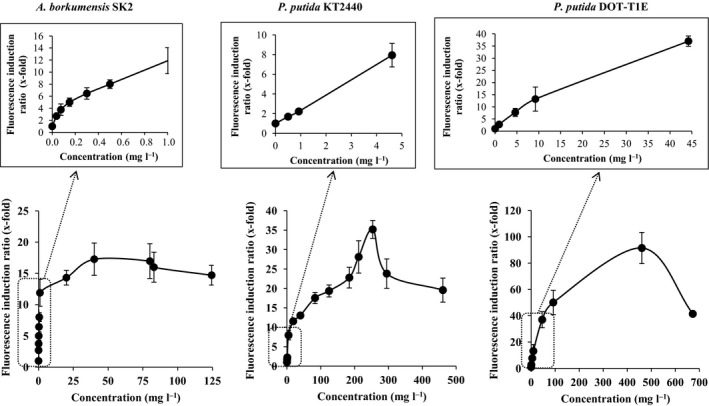
Determination of the detection and saturation limits for pure toluene. *Alcanivorax borkumensis* SK2 (pKST‐1) was cultivated overnight on ONR7a plus sodium pyruvate and 50 μg ml^−1^ of streptomycin as described above, and *Pseudomonas putida* strains containing pKST‐1 were grown on M9 minimal media (Abril *et al*., 1989) plus 5 mM sodium citrate and 150 μg ml^−1^ of streptomycin. Experimental procedures were done as in Fig. 1C. Dotted lines indicate the detection limits. Saturation limits are shown in the upper part of each figure.

The minimum detection limit is described as the inducer concentration at which the fluorescence emitted by the bioreporter in the presence of the inducer is twofold higher than in the absence of inducer. The saturation limit is considered the inducer concentration at which the biosensor reaches its maximum fluorescence emission (Sevilla *et al*., 2015). As shown in Fig. 2, toluene detection limit in *A. borkumensis* SK2 (pKST‐1) was lower than with the two *P. putida* strains (0.04 mg l^−1^ versus 0.9 mg l^−1^). Saturation limits were also lower for *A. borkumensis* SK2 (40 mg l^−1^) than for *P. putida* KT2440 (250 mg l^−1^) and for *P. putida* DOT‐T1E (460 mg l^−1^). The linear range of each biosensor is also different, from 0.04 mg l^−1^ to 1 mg l^−1^ for *A. borkumensis* SK2 (pKST‐1), from 1 mg l^−1^ to 5 mg l^−1^ for *P. putida* KT2440 (pKST‐1) and from 1 mg l^−1^ to 50 mg l^−1^ for *P. putida* DOT‐T1E (pKST‐1) (Fig. 2).

The biosensors limits are in direct correlation with the sensitivity of each strain towards the organic solvent. Solvent extrusion by efflux pumps is one of the main mechanisms of solvent tolerance. In *P. putida* DOT‐T1E, three efflux pumps have been described as responsible for the high solvent tolerance, while in the *P. putida* KT2440 genome, only one of these efflux pumps is present (Rojas *et al*., 2001; Segura *et al*., 2003). Probably because organic solvents are extruded efficiently in *P. putida* DOT‐T1E, the saturation limits are higher for most compounds as those determined in *P. putida* KT2440. There is no information available about efflux pumps in *A. borkumensis* SK2, but the data about its tolerance towards toluene (presented above) suggest lower toluene efflux efficiency in this strain than in *P. putida* strains, explaining the lower detection limits. In all cases, the toluene saturation limits were below the toxic concentration for the two most sensitive strains *A. borkumensis* and *P. putida* KT2440 (that was between 0.1% and 0.2% (v/v) or around 0.87–1.73 g l^−1^). At toluene concentrations higher than the saturation limit, the fluorescence emission decreased, probably because the adverse effects of toluene on the biological membranes and energy consumption (Segura *et al*., 2012).

**Figure 3:**
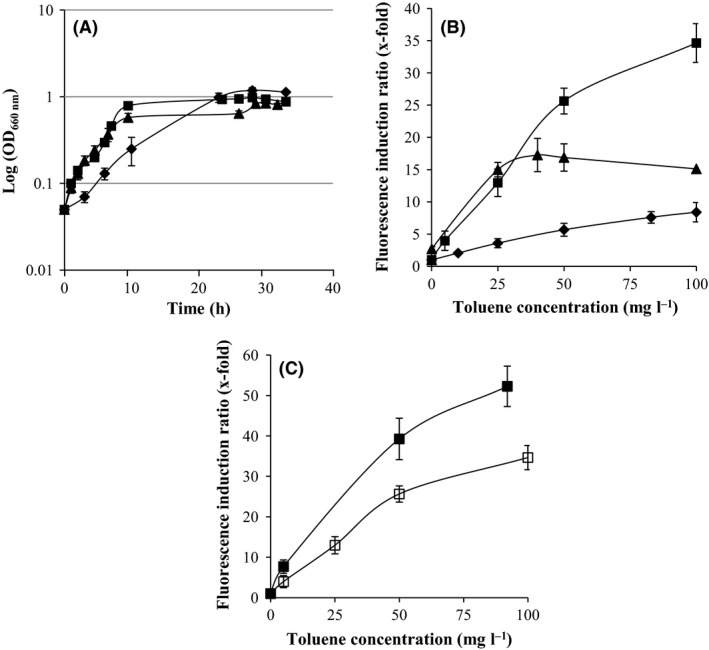
Biosensors performance in marine media. (A) Growth of the strains in marine media with 1% pyruvate (*A. borkumensis* SK2; triangles) and with 5 mM sodium citrate (*Pseudomonas putida* KT2440; diamonds and *P. putida* DOT‐T1E squares). (B) Induction of the three biosensor with toluene in marine media; squares: *P. putida* DOT‐T1E (pKST‐1), diamonds: *P. putida* KT2440 (pKST‐1) and triangles: *A. borkumensis* SK2 (pKST‐1). (C) Performance of *P. putida* DOT‐T1E (pKST‐1) biosensor with toluene as an inducer in marine media (open symbols and dotted line) and minimal media (closed symbols and continuous line).

These experiments were done using M9 minimal media to cultivate the two *P. putida* strains and marine media for *A. borkumensis* SK2. To further demonstrate the importance of the host selection for detection of toxic compounds, we also analysed the performance of our biosensors in marine media. We could not test performance of *A. borkumensis* SK2 (pKST‐1) in M9 minimal media because this strain was not able to grow in this media. *P. putida* DOT‐T1E and *P. putida* KT2440 were able to grow in marine media, although *P. putida* KT2440 grew slower than *P. putida* DOT‐T1E (Fig. 3A). Performance of *P. putida* KT2440 (pKST‐1) was very poor (Fig. 3B), probably because the strain is stressed at high salt concentrations. *P. putida* DOT‐T1E (pKST‐1) showed higher detection limit than *A. borkumensis* SK2 (pKST‐1) (2.5 mg l^−1^ versus 0.04 mg l^−1^) (data not shown). The induction rates of the *P. putida* DOT‐T1E (pKST‐1) biosensor were higher in M9 minimal media than in marine media (Fig. 3C). Therefore, we decided to perform all the following experiments using M9 minimal media when using either of the two *Pseudomonas* strains and marine media when using *A. borkumensis* SK2.

**Figure 4:**
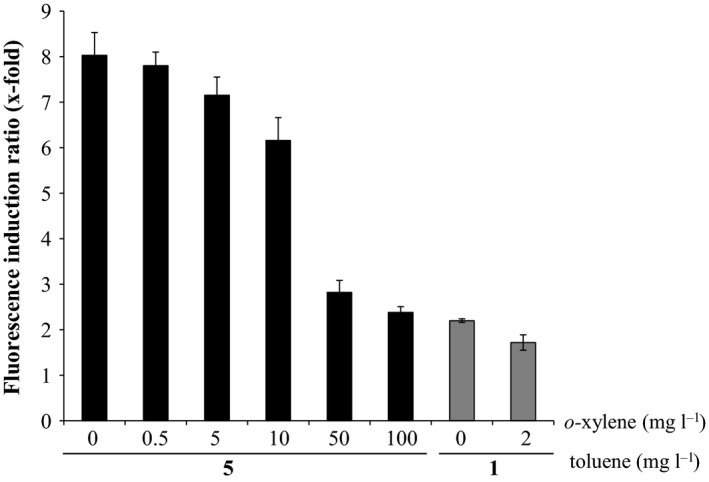
Effect of *o*‐xylene in the response to toluene by *Pseudomonas putida* KT2440 (pKST‐1). Toluene is kept at a constant concentration of 5 mg l^−1^ or 1 mg l^−1^ and increasing concentrations of *o*‐xylene are added.

Biosensors should be specific for the desired compound or family of compounds (Ripp, 2005). Previous results from Lacal *et al*., 2006 have shown that the TodST‐*P*_*todX*_ system was induced by several monocyclic aromatic compounds (toluene and some chloro‐ or fluoro‐toluenes), but they did not determine the induction by other compounds present in oil spills, such as polycyclic aromatic hydrocarbons (PAHs). We tested the specificity of the bioreporter using *P. putida* DOT‐T1E (pKST‐1) as host strain. No induction was observed in the presence of naphthalene (added as a small crystal), 2‐methyl‐naphthalene (added in a glass dipstick into the incubation flask) or phenanthrene (added as a small crystal) (saturating concentrations in aqueous media of 31.6 mg l^−1^, 24.6 mg l^−1^ and 1.6 mg l^−1^, respectively). These results are in agreement with previous experiments of Busch *et al*. (2007) that demonstrated that some compounds with two aromatic rings such as naphthol or 2,3‐dihydroxynaphthalene did not bind to TodS (Busch *et al*., 2007). Experiments were done to test the suitability of these biosensors to detect other BTEX compounds. Biosensors were able to detect benzene, *p*‐xylene and *m*‐xylene in addition to toluene (Table 1); no induction by ethylbenzene was detected at any concentration. Interestingly, saturation limit for *m*‐xylene was similar for the three strains; this, together with the high detection limits, 40, 45 and 70 mg l^−1^ for *P. putida* DOT‐T1E (pKST‐1), *P. putida* KT2440 (pKST‐1) and *A. borkumensis* SK2 (pKST‐1), respectively (Table 1), suggests that this compound is not a good inducer of the system. *O*‐xylene was previously determined to be an antagonist (Busch *et al*., 2007), and when we added different concentrations of *o*‐xylene to a culture induced with 5 mg l^−1^ of toluene, we observed a progressive decrease fluorescence (Fig. 4).

**Table 1:** Saturation and detection limits of the different biosensors with toluene, benzene, *p*‐xylene and *m*‐xylene

	LD	Induction fold	LS	Induction fold	Basal fluorescence
*Alcanivorax borkumensis* SK2 (pKST‐1)					
Toluene	0.04	2 ± 0.31	40	17.01 ± 1.58	90 236 ± 4711
Benzene	0.15	2 ± 0.12	0.5	8.91 ± 0.62	
*p*‐Xylene	1.5	2 ± 0.35	80	16.87 ± 0.27	
*m*‐Xylene	70	2 ± 0.43	125	14.15 ± 0.41	
*Pseudomonas putida* KT2440 (pKST‐1)					
Toluene	0.9	2 ± 0.09	250	35.20 ± 1.8	46 837 ± 7616
Benzene	0.4	2 ± 0.22	100	23.45 ± 1.20	
*p*‐Xylene	25	2 ± 0.04	110	11.50 ± 0.23	
*m*‐Xylene	45	2 ± 0.28	125	9.74 ± 0.48	
*P. putida* DOT‐T1E (pKST‐1)					
Toluene	0.9	2 ± 0.59	460	91.44 ± 10.12	69 022 ± 2769
Benzene	0.2	2 ± 0.14	150	53.55 ± 1.07	
*p*‐Xylene	5	2 ± 0.47	125	39.47 ± 0.78	
*m*‐Xylene	40	2 ± 0.12	125	17.31 ± 1.03	

Concentration data are given in mg l^−1^.

LD: detection limit, concentration when induction ratio is 2. LS: saturation limit, concentration when induction ratio is maximum. Induction fold: relative to the fluorescence of the control without inducer.

Our results demonstrated that the biosensor is specific for monocyclic aromatic hydrocarbons, being able to detect some BTEX compounds, specifically benzene, toluene, and *p*‐ and *m*‐ xylene. The best inducer is benzene, followed by toluene and *p*‐xylene.

This is not the first bioreporter to be constructed for the detection of BTEX; however, many of them have used elements (*lux* or *luc* genes) that require the addition of a secondary exogenous substrate for its activation, such as *lux‐* or *luc‐*based reporters (Applegate *et al*., 1998; Willardson *et al*., 1998; Tecon *et al*., 2010), while GFP‐based bioreporters, such as those described here, do not require the addition of any exogenous compounds (Van der Meer and Belkin, 2010). Although the detection limits of our biosensors are very similar to those reported in the analogous system, *P. putida* F1 (Applegate *et al*., 1998), the saturation limit for *P. putida* DOT‐T1E (pKST‐1) is higher and the saturation limit of *A. borkumensis* SK2 (pKST‐1) is lower than those reported before, demonstrating the importance of the host selection for the detection of toxic compounds.

### Bioreporter validation in contaminated waters

To validate the biosensors, we tested them in different contaminated waters; specifically in waters spiked with petrol (Star Gasoline, CEPSA) or diesel (Star Diesel Oil A+, CEPSA) and in environmental samples from accidental spills. Determination of monocyclic and PAHs in the samples was performed in parallel using a gas chromatography‐mass spectrometer (GC‐MS) (Data S1; Table 2).

**Table 2:** Analysis of BTEX and PAHs content by GC‐MS in environmental samples

	Benzene	Toluene	Ethylbenzene	*m*‐, *p*‐xylene	*o*‐xylene	Total BTEX	Total PAH (2, 3 rings)
Marine water							
Salobreña	0.00	0.00	0.00	0.00	0.00	0.00	0.0045
Motril	0.00	0.00	0.00	0.00	0.00	0.00	0.0057
Messina	0.00	0.00	0.00	0.00	0.00	0.00	0.0105
Gela	0.00	0.00	0.00	0.00	0.00	0.00	0.0117
Edaphic sediments							
Messina	0.00	0.00	0.00	0.00	0.00	0.00	0.0041
Gela	0.00	0.00	0.00	0.00	0.00	0.00	0.0504
Oil and derivates							
Petrol	5248.7250	32 807.6250	8647.1250	16 481.7500	11 175.3750	74 360.6000	153.5490
Diesel	177.8800	604.2850	1082.6650	2363.3400	1823.0950	6051.2650	70.0740
Prestige	0.0405	0.1080	0.0875	0.1298	0.1186	0.4844	0.1938
Crude oil	0.00	0.0461	0.0597	0.0909	0.0777	0.2745	1.2982
Soil 0.01%							
Day 1	0.018	0.106	0.016	0.019	0.031	0.19	nd
Day 3	0.015	0.078	0.015	0.021	0.028	0.155	nd
Day 6	0.009	0.033	0.012	0.018	0.026	0.098	nd
Soil 0.10%							
Day 1	0.026	0.384	0.095	0.111	0.292	0.908	nd
Day 3	0.018	0.166	0.034	0.052	0.132	0.401	nd
Day 6	0.019	0.138	0.028	0.025	0.113	0.322	nd
Soil 0.50%							
Day 1	0.057	1.263	0.592	1.289	2.129	5.329	nd
Day 3	0.026	0.493	0.238	0.477	1.114	2.348	nd
Day 6	0.027	0.377	0.155	0.362	0.668	1.588	nd

Diesel was prepared as a 0.02% solution and petrol as 0.004% in water for GC analysis; concentrations in the table referred to pure diesel or petrol.

Saturated samples (1 g of crude oil and 4 g of Prestige spill oil) were added to the corresponding minimal media and gently agitated overnight in a closed bottle in the dark.

No preparation was needed for Mesina, Gela, Motril and Salobreña water solutions; samples were added directly to the GC‐MS vials. For Gela and Mesina edaphic sediment samples, 5 g of sediment was added to 5 ml of water and agitated overnight in a closed bottle. Samples were analysed after centrifugation to avoid any material in suspension.

In soil experiments, the soil was contaminated with 0.01%, 0.1% or 0.5% (v/w) of petrol; 2 g of soil containing was added to 6.5 ml of water. After vortexing for 1 min, samples were centrifugated at 9500 rpm at room temperature for 10 minutes; 2 ml of the supernatant was transferred to 20 ml tube and water was added to 10 ml total.

Values are given in mg l^−1^.

n.d.: not determined.

None of the biosensors were induced when exposed to 5% and 10% (v/v) waters from Motril and Messina harbours or Salobreña and Gela beaches; no signal was found either for the Messina or Gela edaphic sediments (3 ml of saturated solution was added to 7 ml of culture and incubated for 5 h). Neither was BTEX detected in these samples by GG‐MS analysis (Table 2); therefore, we can conclude that the biosensor is not giving false positives. All the biosensors were induced in the presence of petrol and diesel (Fig. S1). As previously shown with pure toluene, saturation limits were higher in *P. putida* DOT‐T1E (pKST‐1) than in *P. putida* KT2440 (pKST‐1) and *A. borkumensis* SK2 (pKST‐1); detection limits were lower in *A. borkumensis* SK2 (pKST‐1) than in the two *P. putida* strains (Table 3; Fig. S1). It should be pointed out that the equivalent toluene concentration in the detection limit for petrol is lower than for pure toluene; this is related with the presence of other inducers (benzene and xylenes) in the mixture. The equivalent BTEX concentration in detection limit in diesel is always higher than in petrol probably because the ratio between benzene and toluene (best inducers) versus *o*‐xylene (antagonist) is 3.4 in petrol and 0.43 in diesel.

**Table 3:** Saturation and detection limits with different samples

	*Alcanivorax borkumensis* SK2			*Pseudomonas putida* KT2440			*P. putida* DOT‐T1E		
	Petrol	Diesel	Pure toluene	Petrol	Diesel	Pure toluene	Petrol	Diesel	Pure toluene
Saturation limits									
Biosensor saturation limits (% in solution)	0.01	0.8	0.005	0.1	1	0.03	0.23	4.5	0.06
Equivalent BTEX concentration (GC‐MS)	7	48	40	74	60	250	171	272	460
Equivalent toluene concentration (GC‐MS)	3	4	40	33	5	250	75	23	460
Detection limits									
Biosensor detection limits (% in solution)	0.0001	0.05	0.000005	0.0005	0.15	0.0001	0.0008	0.08	0.0001
Equivalent BTEX concentration (GC‐MS)	0.07	3	0.04	0.37	9	0.9	0.59	4	0.9
Equivalent toluene concentration (GC‐MS)	0.03	0.3	0.04	0.15	0.9	0.9	0.24	0.4	0.9

Sensibility limits of the different biosensors; data in mg l^−1^ are obtained on the basis of the determinations by GC‐MS (Table 2).

We also tested the three biosensors in aqueous solutions containing 5% and 10% (v/v) of saturated solutions of crude oil and oil from the Prestige spill (prepared as indicated in Table 2), but none of them induced fluorescence. These samples contained 0.04 mg l^−1^ (crude oil) and 0.11 mg l^−1^ (Prestige oil) of toluene (measured by GC‐MS), a concentration very close to the detection limit of the *A. borkumensis* SK2 (pKST‐1) biosensor. Because *o*‐xylene is an antagonist of toluene in the induction of P_*todX*_ (Busch *et al*., 2007; Fig. 4), we hypothesized that under these conditions, there may be a certain degree of inhibition of the system that accounted for the higher detection limit of the biosensor in these samples when compared with that in pure toluene. Accordingly, 2 mg l^−1^ of *o*‐xylene decreased fluorescence when the system was induced with 1 mg l^−1^ of toluene (Fig. 4).

All these results indicated that biosensors can accurately discriminate between the presence/absence of monocyclic aromatic hydrocarbons in complex mixtures. Although quantification of BTX concentrations in the different samples cannot be as accurately determined as by chemical means, they are useful for quick measurements of pollutants, and a correlation between the intensity of the signal and the amount of monocyclic aromatic hydrocarbons (Fig. S1) provides values of the magnitude of the contamination. Not many of the biosensors reported previously have been tested using environmental samples; the *Escherichia coli luc*‐based bioreporter (Willardson *et al*., 1998) was tested with groundwater samples and samples contaminated with jet fuel although only the detection limits of the biosensor were determined for the groundwater samples (9.2 mg l^−1^ of BTEX). Our *A. borkumensis* SK2 (pKST‐1) biosensor was able to detect 0.07 mg l^−1^ of BTX in samples contaminated with petrol (Table 3). Similarly, Tecon *et al*. (2010) mimicked oil spills in seawaters determining that the minimum toluene equivalent concentration was detected by the *E. coli lux*‐based bioreporter was 7.37 mg l^−1^, 100 times higher than that of the *A. borkumensis* SK2 (pKST‐1) biosensor, 20 times higher than the *P. putida* KT2440 (pKST‐1) biosensor and 12 times higher than *P. putida* DOT‐T1E (pKST‐1).

### Petrol volatilization in soil

**Figure 5:**
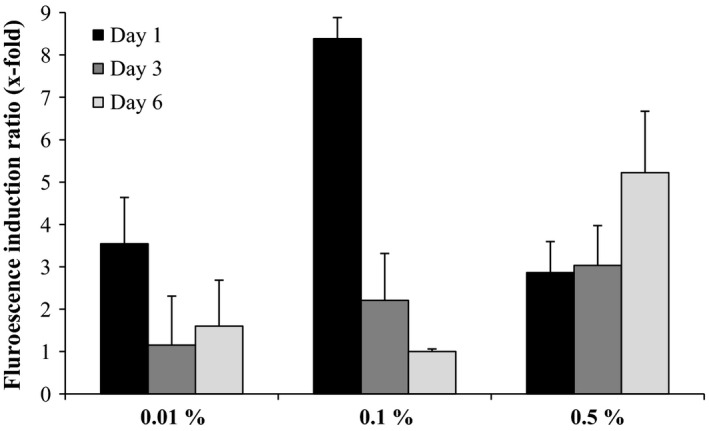
Detection of petrol in soils by *Pseudomonas putida* KT2440 (pKST‐1). A mixture of 59% sand and 41% peat was contaminated with different gasoline concentrations (0.01%, 0.1% and 0.5% of gasoline). Open pots with 100 g of soil were left at room temperature; 1 g of samples was taken at 1, 3 and 6 days of incubation and were added to 10 ml of *P. putida* KT2440 (pKST‐1) culture (OD_660 nm_ ≈ 0.1). After 5 h of incubation at 30°C in an orbital shaker, 2 ml of the culture was centrifuged and fluorescence was measured. Bars indicate the induction fold with the extracts from soils after incubation 1 day with toluene (black), 3 days (dark grey) and 6 days (grey). Error bars mean the standard deviation of three experimental repeats.

We used our *P. putida* KT2440 (pKST‐1) biosensor to evaluate its performance during pollutant elimination in soil. The selection of the biosensor was done on the basis of the good persistence of *P. putida* KT2440 in soils (Molina *et al*., 2000). BTEX are highly volatile compounds (Lim *et al*., 2014), and we decided to analyse the volatilization of these compounds in soil artificially contaminated with 0.01%, 0.1% and 0.5% (v/w) of petrol. As shown in Fig. 5, all the samples gave positive responses. When the soil was contaminated with 0.01% (v/w) of petrol, induction was obtained after only 1 day; on the following days, the amount of toluene (Table 2) was much lower than the detection limit of this biosensor (equivalent toluene concentration 0.15 mg l^−1^; Table 3). When petrol was added to the soil at a concentration of 0.1% (v/w) (Fig. 5), the levels of induction decreased with time, as did the toluene concentration in the samples (Table 2). At day 6, BTEX concentrations were below the limit of detection (LD: 0.37 mg l^−1^; Table 3). Soils polluted with 0.5% (v/w) of petrol showed lower inductions than the expected on days 1 and 3 (Fig. 5). The number of biosensor cells in the culture increased from 10^8^ to 10^9^ cells ml^−1^ after the 5 h of incubation; therefore, we disregarded the possibility of toxicity issues. However, the concentration of *o*‐xylene in the samples after 1 and 3 days is relatively high (2.13 and 1.11 mg l^−1^); in artificial simple systems, at low toluene concentrations (5 mg l^−1^) and using 2 mg l^−1^ of *o*‐xylene, we observed certain inhibition of the fluorescence (Fig. 4) supporting the hypothesis of biosensor inhibition by *o*‐xylene in environmental samples. These issues are not observed at day 6, when the induction of the biosensor showed maximum activity. All these results demonstrate that this biosensor is able to detect BTX in a reliable and simple assay and that it can be useful for rapidly mapping contamination.

To sum up, we have constructed three different biosensors for the detection of monocyclic aromatic compounds which are highly specific for these compounds and that can be used in different environmental samples. Utilization of the *A. borkumensis* SK2 (pKST‐1) biosensor gave the best results detecting lower concentrations of pollutant; however, the low tolerance towards some of these compounds could limit its utilization in heavily contaminated environments. In these cases, *P. putida* DOT‐T1E (pKST‐1) would be the best option due to its high solvent tolerance and the higher saturation limits of the biosensor. For marine environments, *A. borkumensis* SK2 (pKST‐1) would be the best biosensor to choose as salinity will not affect the response of the biosensor. Remarkably, although *P. putida* DOT‐T1E (pKST‐1) performed better in its natural media, it can also be use in marine media if the high organic solvent concentrations recommended the utilization of a high solvent tolerant strain.

Future efforts to miniaturize the biosensors in flow‐cell based systems and the development of systems for the detection of multiple contaminants will allow the monitoring of bioremediation strategies in a fast, easy and economical assay that can be performed on site.

## Conflict of interest

None declared.

## Supporting information


**Fig. S1.** Determination of the detection and saturation limits for petrol (A) and diesel (B) samples.

**Table S1.** Primers and conditions for the amplification of the different components of the BTX bioreporter.

**Data S1.** GC‐MS experiments and Environmental samples.
