# Supplementary material for: New family of biosensors for monitoring BTX in aquatic and edaphic environments
Source: Microb Biotechnol. 2016 Aug 3;9(6):858–67. doi: 10.1111/1751-7915.12394 (PMC5072201; doi:10.1111/1751-7915.12394)
Supplement: Supplementary file 1 — Fig. S1. Determination of the detection and saturation limits for petrol (A) and diesel (B) samples. [file MBT2-9-858-s001.pptx]

## Slide 1
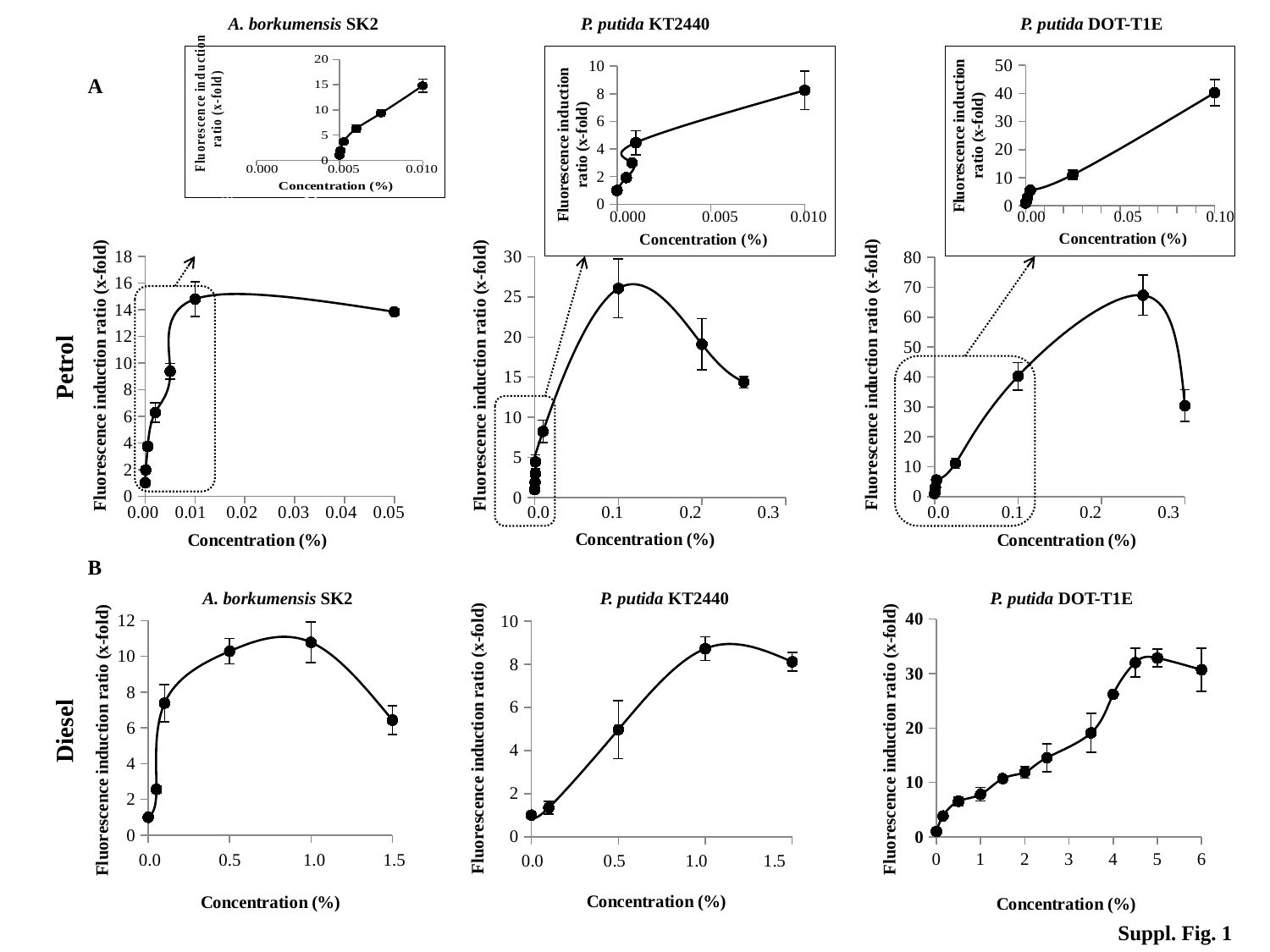

A. borkumensis SK2
P. putida KT2440
P. putida DOT-T1E
### Chart
| Category | |
|---|---|
### Chart
| Category | |
|---|---|
### Chart
| Category | |
|---|---|A
### Chart
| Category | |
|---|---|
### Chart
| Category | |
|---|---|
### Chart
| Category | |
|---|---| 0.000 0.005 0.010
 0.00 0.05 0.10
Petrol
B
### Chart
| Category | |
|---|---|
### Chart
| Category | |
|---|---|
### Chart
| Category | |
|---|---|P. putida DOT-T1E
A. borkumensis SK2
P. putida KT2440
Diesel
 0.0 0.5 1.0 1.5
Suppl. Fig. 1
