# Supplementary material for: New family of biosensors for monitoring BTX in aquatic and edaphic environments
Source: Microb Biotechnol. 2016 Aug 3;9(6):858–67. doi: 10.1111/1751-7915.12394 (PMC5072201; doi:10.1111/1751-7915.12394)
Supplement: Supplementary file 2 — Table S1. Primers and conditions for the amplification of the different components of the BTX bioreporter. [file MBT2-9-858-s002.doc]

**Suppl. Table 1**: Primers and conditions for the amplification of the different components of the BTX bioreporter. Underlined are shown the restriction sites incorporated into the primer for easy ligation. After one denaturalization step of 94ºC for 2 min, 30 amplification cycles were performed at the following conditions: 30 s at 94ºC, 1 min at the hybridization temperature, and 1 min for each kb to be amplified at 72ºC. A final extension of 7 min was performed at 72ºC.

Overlapping PCR conditions for amplifying *P_todX_::gfp* with primers PtodXfor and GFPrev were: one cycle of 94ºC for 2 min, 15 cycles (30 s at 94ºC, 1 min at 50ºC, 1 min at 72ºC) and 25 cycles (30 s at 94ºC, 1 min at 61 ºC, 1 min at 72ºC). Finally, 7 min of final extension were performed at 72ºC.

To construct the pKST-1 bioreporter the DNA fragments containing *todST* and *P_todX_* were amplified using *P. putida* DOT-T1E chromosomal DNA as template and the *gfp* gene was amplified using plasmid DNA (pGreen-TIR from Miller *et al.,* 1997). Overlapping PCR was used to amplify *P_todX_::gfp* using PCR products as template. The *P_todX_::gfp* fragment was cut with *Spe*I and *BamH*I, and cloned into pSEVA438 previously cut with the same enzymes. Ligation was electroporated into *E. coli* DH5α and the resulting plasmid checked by restriction analysis. The resulting plasmid and the *todST* fragment were cut with *BamH*I and *Sac*II and ligated together and transformed into *E. coli* DH5α. The final construction (pKST-1) was checked by restriction analysis and sequencing.

| **Primer** | **Sequence** | **Annealing Tª** | **Amplif. Time** | **Amplicon size** | **Restriction enzyme** |
| --- | --- | --- | --- | --- | --- |
| TodSTfor | CCCCGCGGGGATGATACGAGGGCGTATG | 64ºC | 3 min | 3.5 kb | *Sac*II |
| TodSTrev | AAGGATCCACTATTCCAGGCTATCCTTG |  |  |  | *Bam*HI |
| GFPfor | GGAAGGATTGTAATTATGAGTAAAGGAGAA | 62ºC | 50 sec | 0.7 kb | - |
| GFPrev | AAGGATCCCTATTTGTATAGTTCATCCA |  |  |  | *Bam*HI |
| PtodXfor | AAACTAGTGGTCTGAGGTTTTCATCGAC | 62ºC | 30 sec | 0.3 kb | *Spe*I |
| PtodXrev | TTCTCCTTTACTCATAATTACAATCCTTCC |  |  |  | - |
