# Supplementary material for: New family of biosensors for monitoring BTX in aquatic and edaphic environments
Source: Microb Biotechnol. 2016 Aug 3;9(6):858–67. doi: 10.1111/1751-7915.12394 (PMC5072201; doi:10.1111/1751-7915.12394)
Supplement: Supplementary file 3 — Data S1. GC‐MS experiments and Environmental samples. [file MBT2-9-858-s003.doc]

**GC-MS experiments**

Total BTEX of each solution was determined by solid-phase micro-extraction coupled to a gas chromatograph with a mass-spectrometry detector (SPME-GC/MS). BTEX compounds were analyzed with a 65 µm PDMS/DVB fibre (Supelco, Bellefonte PA, USA), conditioned at 250°C for 0.5 h prior to  use. The fibre was immersed into 20 ml vials filled with 10 ml of sample (Head Space SPME mode) at 30°C during 4 minutes immediately drawn back into the needle and transferred without delay (less than 5 s) into the injection port of the GC (CTC Analytics CombiPal autosampler system). Desorption time 3 minutes (splitless time 3 minutes) at 150°C. The analyses were performed on a Varian Model 450GC coupled to a 240 MS detector. The chromatographic column was a Thermo TG-5SILMS 30 m × 0.25mm × 0.25 μm film. Helium was the carrier gas at 1 ml/min. GC oven program was: initial 45°C (3.5 min.), ramped at 38°C/min. to 80°C held for 4 min., ramped at 75°C/min. to 150°C held 6 minutes. The mass spectra were obtained by Electron Impact Full Scan and SIM mode (45-300 m/z mass scan range). The compounds were quantified by using calibration curves from the corresponding analytical standards. The most probable metabolite structures were based on their retention times, fragment ions of the standard and by using NIST library spectra included in the MS Workstation software 6.9.1.

**Environmental samples**

Water samples were taken from Salobreña beach (Granada/Spain, [36.733262, -3.591925](javascript:void(0))), Motril harbour (Granada/Spain, [36.724116, -3.527764](javascript:void(0))), Messina harbour (Sicily/Italy, [38.190252, 15.558774](javascript:void(0))) and Gela’s water close to its refinery (Sicily/Italy, 37.059541, 14.261727). Edaphic sediments were taken close to Gela’s refinery (Sicily/Italy, 37.059541, 14.261727) and Messina harbour (Sicily/Italy, [38.190252, 15.558774](javascript:void(0)))

Dansk blend crude oil was provided by Fernando Rojo (Centro Nacional de Biotecnología, Spain).

Prestige oil was taken where the oil spill occurred (Ferrol/Spain, [43.488046, -8.322513](javascript:void(0))) and it was provided by Silvia Marqués (Estación Experimental del Zaidín, Spain).

**Bibliography**

Sabirova, J.S., Ferrer, M., Regenhardt, D., Timmis, K.N., Golyshin, P.N. (2006) Proteomic insights into metabolic adaptations in *Alcanivorax borkumensis* induced by alkane utilization. *J. Bacteriol* **188:**3763–3773.
