# Supplementary material for: New family of biosensors for monitoring BTX in aquatic and edaphic environments
Source: Microb Biotechnol. 2016 Aug 3;9(6):858–67. doi: 10.1111/1751-7915.12394 (PMC5072201; doi:10.1111/1751-7915.12394)
Supplement: Supplementary file 4 [file MBT2-9-858-s004.docx]

**Suppl. Fig. 1: Determination of the detection and saturation limits for petrol (A) and diesel (B) samples.**

Detection limits and linear range are shown in the figures; saturation limits are depicted in the small pictures above the figure.
